# External metallic circle in hepaticojejunostomy

**DOI:** 10.1186/1471-2482-4-14

**Published:** 2004-10-06

**Authors:** Erdal Göçmen, Mehmet Keskek, Mesut Tez, Sebat Karamürsel, Mahmut Koç, Mehmet Kılıç

**Affiliations:** 1 Department of 5th Surgery, Numune Training and Research Hospital, Sıhhiye, Ankara, Turkey; 2 Department of Plastic and Reconstructive Surgery, Social Insurances Foundation Hospital, Ankara, Turkey

## Abstract

**Background:**

Biliary-enteric anastomosis especially Roux-en Y hepaticojejunostomy is frequently used for biliary diversion in benign biliary strictures. In this study, we present the results of hepaticojejunostomy with external metallic circle.

**Methods:**

Hepaticojejunostomy with external metallic circle were performed in eight male Sprague-Dawley rats. At the end of the third month, anastomoses were analysed for patency and stability of external circles.

**Results:**

Relaparotomy revealed that all the anastomoses were patent and circles were in original places.

**Conclusion:**

To provide the patency of narrow hepaticojejunostomy anastomoses, external metallic circle can be a good alternative to use of internal stents in suitable cases.

## Background

Although the risk of late bile duct cancer complicating biliary-enteric anastomosis has been well documented [1,2], biliary-enteric anastomosis especially, Roux-en Y hepaticojejunostomy is frequently used for high biliary injuries and for biliary diversion in benign biliary strictures [3]. Among the surgical techniques hepaticojejunostomy yields the most favaroble results [4].

External metallic circle had been used for the end to end choledochocholedocostomy in rats by Tez et al [5]. The patency of anastomosis was higher than conventional primary anastomosis with this device.

The aim of this study was to examine applicability of external metallic circle in hepaticojejunostomy.

## Methods

Eight male Sprague-Dawley rats (Laboratory of Experimental Animals, Hacettepe University Faculty of Medicine, Ankara, Turkey) weighing 250 to 300 g were used. The animals housed under environmentally controlled conditions at 21 ± 2°C and 30% to 70% relative humidity with a 12-hour dark and 12-hour light cycle. Free access to water and standard laboratory food was provided. Before the operations, the rats were fasted overnight and were only allowed free access to water. Guiding Principles in the Care and Use of Laboratory Animals was strictly adhered to at all times together with the recommendations from the Declaration of Helsinki.

## Technique

**Figure 1:**
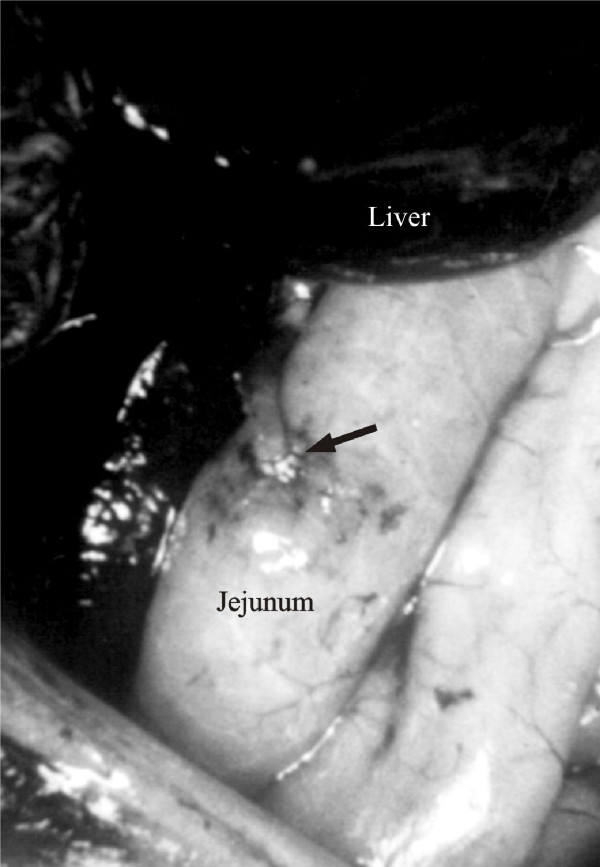
View of hepaticojejunostomy. (Arrows indicates External Metallic Circle)

A surgical microscope (Zeiss, Opmi99, Germany), Codman microsurgical instruments, jeweler's forceps, and 10-0 Ethilon suture were used. Rats were anaesthesized with intramuscular injection of ketamine hydrochloride 100 mg/kg and xylazine 10 mg/kg. Under sterile conditions, a midline abdominal incision was made, and the peritoneal cavity was opened. After the traction of duodenum towards the left, the common bile duct was identified and a complete transection midway between the portal hilus and the duodenum was performed by means of sharp dissection. Proximal end was used for hepaticojejunostomy and distal end was closed by a tie. An opening was made on the wall of the jejunum, wide enough to match the size of the duct at a distance of 4–5 cm from the pylorus. Hepaticojejunostomy was performed by the help of surgical microscope with a silver made external metallic circle. All anastomoses were performed by the same investigator (S.K.). The principle of the technique was to tie the sutures over an external metallic circle 20 to 50 percent greater than the original outer diameter of the bile duct. The circle was handmade from a round-bodied silver wire 0.1 to 0.2 mm thick and 1.0 to 1.2 mm in diameter. The external metallic circle was incorporated at the anastomotic line without any effort to slip it over the cut end of the bile duct. The first suture was placed passing inside the circle and tied over the circle, passing through all layers of duct wall and intestinal wall. The remaining sutures were placed and tied according to the same principles. After completion of sutures, the circle was automatically exteriorised (Figure 1). In a preliminary work, we have performed relaparotomy at the end of third month. Eventhough all the anastomosis were patent, interestingly, we were not able to find any metallic circle around the anastomosis or anywhere else inside the abdomen. Therefore, we modified our technique in this study and placed 2–3 supporting sutures between the circle and jejunal serosa following hepaticojejunostomy anastomosis. These supporting sutures were passing only through the inside of circle and jejunal serosa 4 to 5 mm distant to anastomotic line. At the end of the third month, the rats underwent relaparotomy to investigate the patency of hepaticojejunostomy and stability of circles.

## Results

All anastomoses were completed with five or six sutures. Mean operation time was 30 minutes.

One rat died in the postoperative fourth day. In necropsy, there was anastomotic disruption on the anterior surface of anastomosis and external circle was on the original place.

At the end of third month, relaparotomy was performed on the remaining seven rats. There were no anastomotic dehiscense or biliary leakage. In all animals, there was a good connective tissue mass between the bile duct and jejunum. Dissection of the anastomosis region revealed that all the anastomosis were patent and all the circles were staying in original places.

## Discussion

For the past 10 to 15 years, hepaticojejunostomy has been the method of choice for the treatment of benign biliary stricrures [6,7]. In this study, our aim was to examine the applicability of external circle in hepaticojejunostomy, not comparing the hepaticojejunostomy with or without external metallic circle. Since the first description of injured bile duct repair, many stenting techniques have been used [8]. In clinical practice, there is arguement about the use of internal stents in hepaticojejunostomy. Some authors recommend internal stents when unhealty (ie, ischemic, scarred) and small bile ducts (<4 mm) are found [9]. Braasch [10], Saypol [11] and Cameron [12] have reported high long-term results. when biliary-enteric anastomosis was complimented with internal stent; 80%, 80% and 88% success rates respectively. On the other hand, some authors suggest that biliaryenteric anastomosis can be performed without anastomotic stents. Aust [13], Bismuth [14] and Innes [15] have reported 84%, 86%, 95% success rates respectively when biliary-enteric anastomosis was performed without using any stents and they suggest that a stent may promote fibrosis of the anastomosis due to constant irritation of ductal mucosa. Thus, transanastomotic stents appear to have little impact on outcome and probably should not be used routinely. However, stents still may be useful in selected cases in which poor outcome is considered preoperatively or intraoperatively.

In a previous study of us [5], we showed that end to end biliary anastomosis with an external metallic circle had the advantage of shorter operating time and lower bile leakage rate compared to primary microsurgical anastomosis. And alkaline phosphatase levels were also found to be significantly lower for end to end biliary anastomosis with external metallic circle. This results directed us to search the applicability of external metallic circle in narrow hepaticojejunostomy anastomoses. Therefore, we designed a study to perform end to side hepaticojejunostomy with external metallic circle. During the relaparotomy performed at the end of third month, we found all the anastomosis were patent but we were not able to find circles in original places except in one rat. Later on, in this study, we modified our technique and added 2–3 supporting sutures between the circle and jejunal serosa. Relaparotomy revelad all the anastomosis were patent and circles were still in place.

## Conclusion

We think that external metallic circles are also applicable to end to side hepaticojejunostomy anastomosis, should the extra sutures were placed between the circle and jejunal serosa neighbouring the anastomotic line following the completion of anastomosis. To provide the patency of narrow hepaticojejunostomy anastomoses, external metallic circle can be an alternative to use of internal stents in suitable cases.

## Competing interests

The authors declare that they have no competing interests.

## Authors' contributions

EG designed the study, performed the operations and prepared the manuscript. MK, MT, and MKı participated in performing the operations. MKo participated in the design of study and coordination. SK performed the microsurgical anastomosis. All authors read and approved the final manuscript.

## Pre-publication history

The pre-publication history for this paper can be accessed here:


